# What Do We Know about the Diets of Pacific Islander Adults in Papua New Guinea? A Scoping Review

**DOI:** 10.3390/nu16101472

**Published:** 2024-05-13

**Authors:** Alyse Davies, Juliana Chen, Hannah Peters, Alex Lamond, Anna Rangan, Margaret Allman-Farinelli, Shelina Porykali, Robin Oge, Hans Nogua, Bobby Porykali

**Affiliations:** 1 Discipline of Nutrition and Dietetics, Susan Wakil School of Nursing and Midwifery, Faculty of Medicine and Health, The University of Sydney, Sydney, NSW 2006, Australia; alyse.davies@sydney.edu.au (A.D.);; 2 Charles Perkins Centre, The University of Sydney, Sydney, NSW 2006, Australia; 3 School of Health Sciences and Social Work, Griffith University, Gold Coast, QLD 4222, Australia; 4 National Capital District Provincial Health Authority, Port Moresby 121, Papua New Guinea; 5 Port Moresby General Hospital, National Capital District, Port Moresby 121, Papua New Guinea; 6 Aboriginal and Torres Strait Islander Health Program, George Institute for Global Health, Sydney, NSW 2000, Australia; bporykali@georgeinstitute.org.au; 7 Faculty of Medicine, School of Population Health, University of New South Wales, Sydney, NSW 2052, Australia

**Keywords:** Papua New Guinea, Pacific Islander, diet, nutrition, food, Healthy Islands

## Abstract

While a large proportion of the population in Papua New Guinea (PNG) maintain a subsistence lifestyle, exposure to modernisation and industrialisation since European contact has influenced a transition towards Western diets. This review aimed to scope and summarise the published research on dietary intake among Pacific Islander adults in PNG. Four electronic databases and grey literature were searched. Two reviewers completed the screening and data extraction. Fourteen studies were included from the Highlands (*n* = 7), Southern (*n* = 5), Momase (*n* = 1) and both the Highlands/Southern region (*n* = 1). No studies were from the Islands region. The majority of the studies were published prior to the year 2000 (*n* = 9). Geographical region and degree of urbanisation had an impact on dietary intake. Urban areas reported higher intakes of energy, protein and fat compared to rural areas. In the Southern region, a variety of foods, including sago, taro, kaukau, cooked banana, coconut and cassava contributed to energy intake, while kaukau was the main energy and protein source in the Highlands. The main foods contributing to protein in the Southern region were fresh fish, land animals and purchased animals. This review highlights an evidence gap regarding dietary intake research. Within the context of international initiatives, there is an urgent call for research aimed at understanding the social and cultural contextualisation of dietary behaviours in PNG.

## 1. Introduction

Papua New Guinea (PNG) is renowned for its diversity, being abundant in resources, natural surroundings, cultures and languages. With a population of 10 million as of 2022 [1], PNG is the largest Pacific Island nation. The population is predominately Melanesian, and while there are 800 Indigenous languages, the common language is Tok Pisin (Pidgin English). The country is classified into four regions: Highlands, New Guinea Islands, Momase and Southern, with each of the regions having different environments, cultures and needs. PNG gained independence from Australia on 16 September 1975. Since then, urbanisation has increased due to the trading of their rich natural resources acting as a catalyst, leading to economic growth and increased accessibility to the global food supply.

While a large proportion of the population lives in rural environments and maintains a subsistence lifestyle, those living in urban areas, such as Port Moresby, Lae and Madang have increasingly been exposed to modernisation and industrialisation since European contact. There is also a shift in individuals moving from rural areas to urban areas in pursuit of education, employment, and business opportunities. In urban areas of PNG, over 90% of dietary intake is sourced through purchases from local stores and markets [2]. Nutrition transition is the concept used in the literature to describe the evolving dietary patterns within societies, marked by a shift from traditional, locally sourced diets to more processed and ultra-processed foods and beverages, which are less nutrient dense and high in deleterious nutrients, such as saturated fat, added sugar and salt [3,4,5]. The nutrition transition has also been accompanied by lifestyle changes, including less physical activity and loss of farming practices as a result of urbanisation [6]. This shift has implications for public health, particularly the increased risk for non-communicable diseases (NCDs). However, nutrition transitions are poorly captured in low- and middle-income countries (LMICs) [4].

PNG is experiencing a rapid increase in NCDs and their associated risk factors due to ongoing economic and social shifts, as well as nutrition transition, placing additional pressure on the already compromised healthcare system [7]. PNG is classified as a LMIC by the World Bank criteria [8]. LMICs carry the highest burden of disease, where around 80% of NCD-related deaths and premature deaths occur [9,10]. The four main NCDs include cardiovascular disease (such as myocardial infarction and stroke), cancers, chronic respiratory diseases (such as chronic obstructive pulmonary disease and asthma) and diabetes [11]. Modifiable behavioural risk factors that increase the risk of NCDs include smoking, excessive alcohol consumption, physical inactivity and an unhealthy diet [11]. There is also a high prevalence of betelnut chewing with lime and mustard stick in PNG, which constitutes a significant risk factor for oral cancer [12]. Metabolic risk factors include overweight and obesity, hypertension (elevated blood pressure), hyperglycaemia (high blood glucose levels) and hyperlipidaemia (high levels of cholesterol in the blood) [11].

The Healthy Islands initiative embodies a vision that promotes health protection and activities for health promotion across the Pacific Islands countries and territories [13,14]. The PNG government supports the Healthy Islands concept and has also published a national nutrition policy (2016–2026) [15], a national food security policy (2018–2017) [16] and the National Health Plan (2021–2030) [2]. The five key result areas (KRAs) in the National Health Plan include the following: KRA. 1: promoting healthier communities through effective engagement; KRA. 2: working together in partnership; KRA. 3: increasing access to quality and affordable health services; KRA. 4: addressing disease burdens and targeted health priorities; and KRA. 5: strengthening health systems. The overall vision of the plan is “a healthy and prosperous nation where health and wellbeing are enjoyed by all”.

In light of the nutrition transition and the PNG government’s commitment to the Healthy Islands initiative and its national health strategies, a deeper understanding of the diets of Pacific Islander peoples in PNG in terms of their energy intake, macronutrients, micronutrients and food groups is critical for planning future interventions and guiding research directions aimed at addressing the growing burden of diet-related diseases. A scoping review was selected to identify and map all relevant peer-reviewed and grey literature to understand the gaps in knowledge on this topic [17]. This review aimed to scope and summarise the research on dietary intake among Pacific Islander adults or households in PNG.

To contextualise this research in the context of PNG and nutrition, a positionality statement is important. Four members of the research team (R.O., H.N., B.P., S.P) have heritage from PNG and proposed the research question based on their professional, research and clinical experiences, which include witnessing the nutrition transition and subsequent high rates of diet-related diseases. Residing in PNG, R.O. and H.N. are specialist medical doctors. B.P. is a senior lecturer and senior researcher fellow with expertise in population health, while S.P. is a diet aid and student dietitian (Honours). M.A-F. and J.C. have expertise in public health nutrition and have worked on projects in New Caledonia. A.D. and A.R. and M.A-F have expertise in dietary assessment and A.D. has family ties to PNG. H.P. and A.L. were student dietitians with a strong interest in global health. 

## 2. Materials and Methods

### 2.1. Protocol and Registration

The protocol for this scoping review was developed and published on the Open Science Platform, (https://osf.io/h6t5m/ (accessed on 10 May 2023)). The findings were reported according to the Joanna Briggs Institute methodological guidance for scoping reviews and PRISMA Extension for Scoping Reviews [18,19].

### 2.2. Inclusion Criteria

#### 2.2.1. Participants

Studies including Pacific Islander adults from any region within PNG were included. Studies where Papua New Guineans did not make up 50% of the sample population, or where the data could not be separated from other populations, were excluded. Studies including children only were excluded, but household studies or village setting studies with children were included.

#### 2.2.2. Concept

Studies that considered traditional or contemporary dietary intake of Pacific Islander adults in PNG were included. Any method of measuring dietary intake, such as via quantitative, qualitative and mixed methods were considered. Studies that only reported on food availability or insecurity, including sales, import and export data, were excluded due to their inability to reflect dietary intake. Studies exclusively reporting on supplement or alcohol consumption were also excluded.

#### 2.2.3. Context

This review included papers based in any geographical region within PNG; the Highlands, New Guinea Islands, Momase and Southern. Both published scientific literature and grey literature were included. Diets that failed to capture usual intake, such as during COVID-19 or fasting, were excluded. Papers reporting on eating disorders or rapid weight loss diets were excluded.

### 2.3. Types of Sources

All primary study designs that focused on dietary intake were considered for inclusion. Government reports and websites were also included. Reviews and meta-analyses, protocols, conference abstracts, theses and editorials were excluded. Relevant review references were screened to capture any additional studies. Where the full text was not available, studies were excluded.

### 2.4. Search Strategy

A full search strategy was developed by the researchers (H.P., A.L., A.D. and B.P.) and an experienced librarian (M.C.) using a combination of MeSH headings and keywords found in titles and abstracts. The initial search was conducted using MEDLINE to identify relevant MeSH headings and keywords. These terms were adapted for the additional databases. There was no publication date limit set due to the limited existing published research and studies were limited to the English language. The full search was conducted in March 2023 using Ovid (Medline, Embase, and Global Health) and Scopus. Grey literature sources including Google Scholar and government sites were also searched. The search strategy for MEDLINE is presented in Supplementary Table S1.

### 2.5. Selection Process

**Figure 1:**
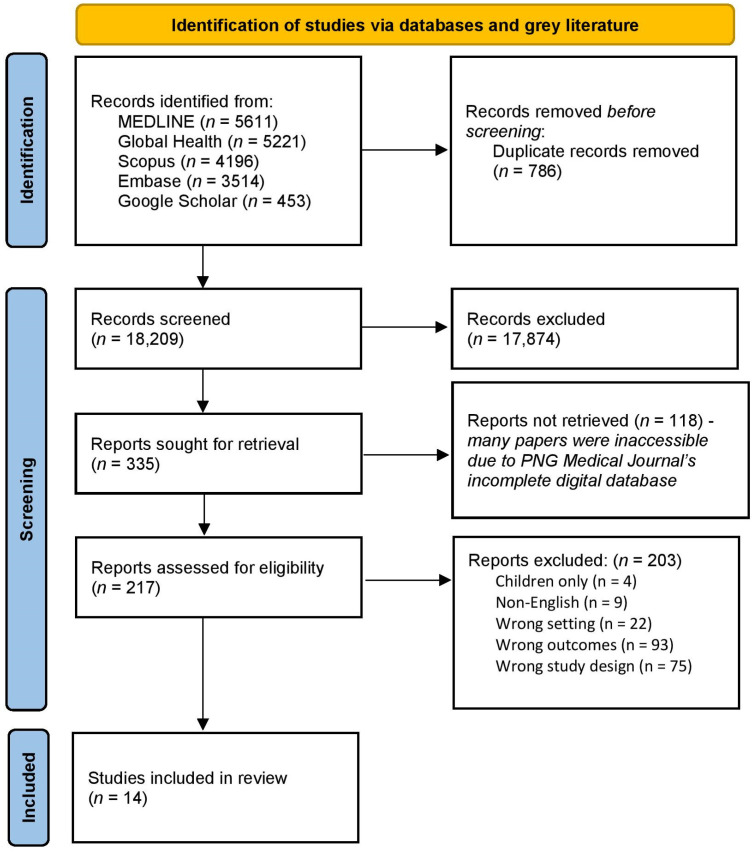
PRISMA flow diagram of record identification and study selection for a scoping review of the diets of Pacific Islander adults in Papua New Guinea (PNG).

Publications that were identified through the full search strategy were imported into EndNote 20 (Clarivate Analytics, Philadelphia, PA, USA) for screening, and duplicates were removed. The titles and abstracts were screened by two independent reviewers (H.P and A.L) against the review inclusion criteria, using Covidence (Veritas Health Innovation, Melbourne, Australia, https://www.covidence.org/ accessed on 09 March 2023). Studies that satisfied the inclusion criteria in the first screening continued to second stage screening, where the full text was used to consider inclusion by two reviewers (H.P and A.L). Any disputes were reviewed by a third independent reviewer (A.D and B.P). The search results are presented in an adapted PRISMA flow diagram [20] (Figure 1).

### 2.6. Data Extraction and Charting

The data were extracted using standardised data charting based on the framework for scoping reviews [18,21]. The following details were extracted: first author, publication year, study design, region/setting, population, data collection date, dietary assessment method(s), additional information for data collection, interview language, data source for foods, primary (energy, nutrients and foods) and secondary (socio-cultural and economic factors of eating) outcome measures.

### 2.7. Synthesis of Results

The results were presented in both tabular and written form, with an accompanying narrative summary to describe the dietary intake among Pacific Islander adults or households in PNG.

## 3. Results

### 3.1. Search Results

Databases and grey literature searching identified a total of 18,995 records. A total of 786 duplicates were removed. Title and abstract screening were performed on the records, and 17,874 irrelevant records were excluded, leaving 335 for full-text screening. An additional 118 reports were excluded due to the full text being unavailable, leaving 217 reports. Full-text screening excluded a further 203 reports, resulting in 14 studies identified for inclusion in the review. The PRISMA flow diagram details the selection process (Figure 1).

### 3.2. Study Selection and Characteristics

The study selection and characteristics are presented in Table 1, with 14 publications included from databases and grey literature sources [22,23,24,25,26,27,28,29,30,31,32,33,34,35]. The publications covered a 48-year time period, from 1969 [22] to 2017 [35]. Three studies were cross-sectional [23,27,35], one was a case-control study [30], while the remaining studies did not report their study design [22,24,25,26,28,29,31,32,33,34]. Seven studies were conducted in the Highlands [22,23,24,28,31,32,34], five in the Southern region [25,26,29,30,35], one in Momase [27] and one in the Highlands/Southern region [33]. No studies were conducted in the New Guinea Islands. The sample population included individuals [22,23,24,25,28,29,30,32,33,34,35], households or families [22,24,27,28,31,35] and villages [25,35]. Both genders were included in 11 studies [22,23,24,25,26,28,30,31,32,33,34], two studies included females only [27,35], and one included males only [29]. The dietary assessment methods varied across studies, including weighing food [22,23,24,25,26,28,29,32,33,34], food recalls [22,24,25,26,27,29,30,32,33,34], food frequency questionnaires [30,34], lists [27], interviews [28], surveys [31,35] and field observations [27,31]. Additional details on dietary assessment methods and data collection can be found in Supplementary Table S1. Six studies reported the interview language [24,27,28,31,32,33] being a local language or the common language, Tok Pisin. Of the 14 included studies, 11 reported on dietary intake (energy and nutrients) [22,23,24,25,26,27,29,30,32,33,34], 12 reported on food sources [22,23,24,25,26,27,28,29,30,31,34,35], and 10 on the socio-cultural and economic context of eating [22,23,24,26,27,28,30,31,33,34]. Eleven studies reported the data sources for foods [22,23,24,25,26,27,29,30,32,33,34]. Eight studies reported comparisons of dietary data with FAO/WHO recommendations [24,25,26,27,29,32,34,35], recommended dietary allowances for a New Guinea native [22] or intakes from Australia [30]. 

### 3.3. Dietary Data

#### 3.3.1. Energy

Energy intake was reported in ten studies, four in the Highlands [22,23,24,32], four in the Southern region [25,26,29,30], one in Momase [27] and one study reporting on both the Highlands and Southern regions [33]. In the Highlands, the reported mean daily energy intake ranged between 9623 and12,230 kJ for males [23,24,33] and between 7406 and 10,530 kJ for females [23,24,33]. One study was not included in the above energy intake range, as it provided data for two females (one lactating) within a household [22]. One study in the Highlands reported a higher mean daily energy intake in flat wetland areas compared to dry hilly areas, respectively, for both males (15,040 kJ vs. 9720 kJ) and females (13,270 kJ vs. 9140 kJ) [32]. In the Southern region, the reported mean daily energy intakes ranged between 5700 and 14,866 kJ [25,26,29,30,33] for males and between 9620 and 10,500 kJ [26,30,33] for females. One study in the Southern region [26] reported lower energy intakes in the year 1975 compared to 1984 (non-working and working) for both males (5700, 7480, 9370 kJ) and females (5630, 6240, 7300 kJ), respectively. Studies in the Southern region reported higher energy intakes in urban areas [30,33] for males (11,500–11,650 kJ) and females (9620–10,500 kJ) compared to males in a rural areas [29] (8660–8880 kJ). One study reported on females in the Momase region [27], with Grass County yielding the highest energy intake data, compared to Wosera and Middle Sepik, respectively (11,820, 10,330, 9652 kJ) (Table 2).

#### 3.3.2. Fibre

Fibre intake was reported in two studies, both in the Southern region [25,30], with the mean daily fibre intake ranging from 10 to 22 g/day (Table 2).

#### 3.3.3. Protein

Protein intake was reported in ten studies, five in the Highlands [22,23,24,32,34], four in the Southern [25,26,29,30], one in Momase [27] and one reporting on both the Highlands and Southern regions [33]. In the Highlands, the reported mean daily protein intake ranged from 25 to 58 g [23,24,32,33,34] and 6%E [24] for males, and from 20 to 70 g [22,23,24,32,33,34] and 6%E [24] for females. In the Southern region, the reported mean daily protein intake ranged from 18 to 96 g [25,26,29,30,33] and 3–14%E [29,30] for males, and from 27 to 93 g [26,30,33] and 15%E [30] for females, with the higher %E from protein reported in a study conducted in an urban area [30], compared to a rural area [29]. In the Momase region, the mean daily protein intake ranged from 54 to 66 g [27] (Table 2).

#### 3.3.4. Carbohydrate

Carbohydrate intake was reported in five studies, one in the Highlands [23] and three in the Southern region [25,29,30]. In the Highlands [23], the mean daily carbohydrate intake was 540 g in males and 410 g in females. In the Southern region, the reported daily carbohydrate intake ranged from 427 to 776 g [25,29,30] and 59%E [30] for males, and 385 g [30] and 58%E [30] for females. No studies reported carbohydrate intake in the Momase region (Table 2). 

#### 3.3.5. Fat

Fat intake was reported in seven studies, two in the Highlands [23,32], four in the Southern region [25,26,29,30] and one study reporting on both the Highlands and Southern regions [33]. In the Highlands, the reported mean daily fat intake ranged from 6 to 18 g [23,32,33] in males and from 7 to 31 g [23,32,33] in females. In the Southern region, the reported mean daily fat intake ranged from 3 to 82 g [25,26,29,30,33] and 23%E [30] for males, and from 3 to 84 g [26,30,33] and 23%E [30] for females. One study in the Southern region [26] reported lower fat intakes in the year 1975 compared to 1984 (non-working and working), for both males (3, 6, 22 g) and females (3, 14, 16 g), respectively. Studies in the Southern region reported higher mean daily fat intakes in urban areas [30,33] for males (71–82 g) and females (65–84 g) compared to males in rural areas [29] (12–15 g). No studies reported fat intake in the Momase region (Table 2).

#### 3.3.6. Micronutrients 

Micronutrient intake was reported in three studies, one from the Highlands [22] and two from the Southern region [26,29]. The micronutrients reported included thiamine, riboflavin, niacin, vitamin A, vitamin C, iron and calcium.

### 3.4. Food Sources of Energy and Protein 

#### 3.4.1. Contribution to Energy Intake

The contributions of food to energy intake were reported in seven studies, four in the Southern region [25,26,29,30] and three in the Highlands region [22,23,24] (Table 3). In the Southern region, garden crops [25], coconut [25], sago [25,29], taro [26], kauaku [26], plantain [29], cooked banana [30] and cassava [30] were important energy sources. Store foods and imported rice were the main energy sources for workers [26], and imported refined carbohydrate sources were reported in urban areas (white rice, bread and rolls) [30]. Kaukau (sweet potato) contributed the most energy to diets in the Highlands, accounting for 53–90% of the total energy intake [22,23,24]. A common snack, sugarcane, contributed between 10–24% of the total energy intake [22], and cereals and grains (rice, flour and maize) contributed 22% [24].

#### 3.4.2. Contributions to Protein Intake

The contributions of foods to protein intake were reported in seven studies, four in the Highlands [22,24,31,34] and three in the Southern region [25,26,30] (Table 3). In the Southern region, land and purchased animals were large contributors to protein intake [25,26]. Fresh fish contributed 28% of the daily intake in an urban area [30]. Taro and kaukau were important plant sources of protein, while animal sources such as tinned meat and fish contributed to the protein intakes of workers [26]. Kaukau was the highest contributor to protein intake in the Highlands [22,24,31]. Trade store foods (e.g., supermarket foods), cereals and grains (rice, flour and maize) and peanuts were also common foods that contributed to protein intake in the Highlands [24,31,34].

### 3.5. Socio-Cultural and Economic Factors of Eating

Ten papers reported contextual information: six in the Highlands [22,23,24,28,31,34], two in the Southern region [26,30], one Momase [27] and one in both the Highlands and Southern region [33]. Four studies conducted in the Highlands [22,23,28,31] described meal timing, and it was reported that two meals were consumed per day (morning and evening), with the midday meal consisting of snacks. Morning meals contained an average of two foods, and the evening meal consisted of four [28]. Evening meals are often shared with friends and family [23]. Mumus (traditional underground cooking) are common for social occasions [22]. Social changes among younger generations have occurred in response to European contact [23]. Seven studies reported an influence of cash cropping and involvement in the cash economy [23,26,27,28,30,33,34], with the main sources of income being cattle and coffee.

## 4. Discussion

This scoping review summarised research on dietary intake among Pacific Islander adults in PNG. The 14 included studies reported on energy intake, nutrients and frequently consumed foods, but most were published before the year 2000. It was found that urban areas tended to have higher intakes of energy, protein and fat compared to rural areas. Working individuals had higher energy intakes compared to non-workers, and store foods and imported rice were some of the workers’ main energy sources. In the Southern region, a variety of foods, including sago, taro, kaukau, cooked banana, coconut and cassava, contributed to energy intake, while kaukau was the main energy and protein source in the Highlands region. A lower percentage of energy from protein for both males and females was reported in the Highlands (6%) compared to the Southern region (14–15%). This percentage was lower than the acceptable macronutrient distribution range for protein, which falls between 15–25% [60]. This needs to be interpreted with caution, as it may not reflect usual intake, given the availability of protein sources and the social and cultural contexts of eating. The main foods contributing to protein intake in the Southern region were fresh fish, land animals and purchased animals, with workers consuming tinned meat. There was minimal reporting on micronutrient intake across the included studies.

Proper nutrition is important for preventing disease [61]. While the PNG government’s 2021–2030 National Health Plan recognises nutrition as a determinant of health and includes nutrition in their KRAs [2], there is a scarcity of contemporary dietary intake data amongst the PNG population. Most studies identified in this scoping review were published prior to the year 2000 and, therefore, are not reflective of the current food environment or the recent dietary changes, particularly in urban areas experiencing the nutrition transition [62]. It is important to understand the social and cultural contextualisation of dietary behaviours in PNG. As a collective society with rich social and cultural practices, food is an integral part of the culture. Examples include the following practices: mumu (traditional underground cooking), where families and communities work together to prepare, cook and consume traditional foods; sing sings (cultural dance celebrations), where different communities share their traditional foods alongside songs and dance; haus krais (places of customary mourning), with ceremonies that can last for weeks, where relatives, friends and colleagues pay their respects, share foods and eat together, offer gifts (which include foods) and financial contributions to show unity and solidify cultural ties and friendships. Furthermore, being a Christian nation, many social eating occasions occur around religious gatherings. These social and cultural traditions, practices and behaviours strongly influence the dietary intake in PNG, and there is a paucity of evidence documenting the contemporary social and cultural context of eating, including how Western foods are being introduced into cultural practices of eating.

Cash cropping and entry into the cash economy were topics of discussion in many studies included in this review, as many were published between the 1980s and 1990s. Cash cropping refers to the practice of cultivating crops primarily for sale in the market, rather than for subsistence or personal consumption. The shift towards cash cropping has been influenced by various factors, including market demand, government policies, and the desire to participate in the cash economy. It has been identified that the money earned from cash crops is likely used to purchase imported store-bought foods such as bread, rice and tinned meat or fish [27,30]. The early studies included in this review reported that these foods were beneficial, particularly for providing protein and energy to children, addressing the issue of malnutrition [28,30]. However, it was also acknowledged that the transition to predominately imported store-bought foods may increase the future prevalence of NCDs [30]. This is now documented, with a systematic review published in 2020 suggesting that the prevalence of NCDs (particularly type 2 diabetes) and the associated risk factors have increased over time [63]. A 2023 Lancet publication on trends and disparities of NCDs in the Western Pacific region reported that PNG had the highest prevalence of diabetes, at 17% in 2021 [64]. The Southern region, specifically in the Central Province and Port Moresby, tends to have higher rates due to longer exposure to modernisation and changes in diet and physical activity patterns [63]. However, there are limited dietary intake studies for the Southern region. To address this growing burden of NCDs, which is at the forefront of the global agenda, it is critically important to conduct contemporary dietary intake research to understand dietary composition and portion sizes. Funding and resource allocation should be prioritised for such research in this area. This fundamental information is needed to help guide future health promotion initiatives and disease prevention strategies, which is the focus of Healthy Islands, an international scale initiative developed by the World Health Organization. Promoting health in island communities contributes to global health security and supports the broader agenda of achieving the United Nations Sustainable Development Goals (SDG), e.g., zero hunger (SDG1), good health and wellbeing (SDG3) and partnerships for the goals (SDG17) [65,66]. The Healthy Islands initiative has reported challenges regarding barriers to implementation due to contextual differences in health responses [14]. To provide direction for good health for the most populous Pacific Island Nation, highlighted in this review was the importance of understanding the social and cultural context of eating, which also has wider implications for NCD prevention. Furthermore, the holistic approach Healthy Islands has towards a unified vision across all Pacific Islands could serve as a model for other LMICs, particularly in terms of sharing resources and methods of assessing diet to reduce NCDs.

The geographical location of PNG significantly shapes its dietary patterns, reflecting the diversity of landscapes across coastal regions, highlands and tropical environments. In the Southern region, diets are notably influenced by the abundance of seafood, with fresh fish contributing 28% of the daily protein intake. The Highlands region is characterised by the cultivation of staple crops, with kaukau contributing between 53 and 90% of energy and between 33 and 43% of protein. This geographical diversity, intertwined with cultural practices, not only shapes the availability and consumption of various food items but also gives rise to unique and region-specific dietary patterns. While fresh market food in urban areas is readily available, accessible and, arguably, more affordable, it appears that store-bought food may be preferential. This warrants further research and public health nutrition education campaigns promoting healthy food choices, as well as education around the detrimental health impacts of processed foods, high in saturated fat, added sugars and salt and ultra-processed foods. Moreover, in urban, compared to rural populations, research highlights a different dietary trend, characterised by higher intakes of energy, protein and fat, whereby the fat intake is predominantly saturated [30]. The evidence suggests that there is an association between saturated fat and the risk of cardiovascular disease [67]. To reduce the risk of chronic disease, the total fat should be between 20 and 35% energy, while saturated fat intake should be limited to no more than 10% of the total energy [60]. While the PNG population is at risk of cardiovascular disease [68], there is insufficient evidence to quantify the current prevalence [63], and it has been suggested that longitudinal studies are needed to monitor changes in rapidly changing societies such as PNG [68].

Being overweight or obese increases the risk for chronic disease, and it is a growing problem in PNG, with 29% of women and 19% of men considered to be obese in 2022 [69]. With the limited progress towards achieving the diet-related NCD targets in PNG, the national nutrition policy aims to prioritise interventions to prevent and control overweight and obesity. Despite this focus, there is a lack of nutrition programs in the literature specifically addressing diet-related NCDs and their associated risk factors in PNG [70]. Nutrition education is a key element of nutrition programs, as it enhances the knowledge, skills and behaviours related to nutrition [71]. Given that PNG is experiencing rapid transformation due to economic development and an increased reliance of the global food supply, messaging around nutrition and health for the broader population is necessary. While nutrition knowledge and education have been recognised as important factors in the PNG national food security policy [16], recent efforts have been initiated to introduce nutrition education in schools [72]. However, there needs to be a life-staged approach that provides education in primary, secondary and tertiary settings. The absence of such an approach poses a challenge to driving meaningful population change in the realm of nutrition and diet-related health outcomes in PNG.

Dietary guidelines are tools that provide public food and nutrition advice to the general public to promote overall health and prevent chronic disease [73]. The guidelines typically describe socially and culturally appropriate dietary patterns supporting individuals to consume food groups in proportions that allow all nutrient needs to be met [74]. Dietary guidelines also provide context for dietary intakes, allowing benchmarks to measure the success of diet and lifestyle interventions and helping to monitor changes over time [74]. This tool is used in many countries worldwide, with each developing guidelines optimised for their nations, considering food availability, preferences, needs and social and cultural factors [73]. PNG published a ten-year national nutrition policy [15], which aims to focus on and build momentum towards improved nutrition status; however, it has yet to disseminate the dietary guidelines for the general population.

It is important to comment on the dietary assessment methods to ensure the credibility and relevance of the findings. A previous study documented the lack of standardised tools for measuring diet in LMICs [4]. The majority of studies in this review used a combination of methods, mostly involving weighing foods, food recalls and direct observations. We specifically selected the wording of ‘weighing foods’, rather than the conventional ‘weighed food records’, given the differences in approaches to data collection stemming from social and cultural ways of eating. The conventional weighed food record involves individuals weighing and recording all foods and beverages at the time of consumption in a record booklet or app. The ‘weighing of food’ in the studies from PNG was performed by the investigator for two meal occasions (morning and evening). The investigator would arrive at the household at sunrise to weigh the morning meal. Upon the return of the day’s harvest, the food would be sorted by the investigator and weighed prior to the evening meal. The evening meal is usually prolonged over several hours, during which food is continually cooked and eaten. On occasions, the investigator would visit household gardens to document the size and number of crops. Snacking was common during the day, and these data were usually collected via recall, with quantities estimated or by weighing a similar portion of the item. The method of a 24 h recall was also mentioned; however, the procedure for data collection, such as the use of the multiple pass method, was unclear. With the advancement of technology, it may be possible to consider apps for the collection of dietary data. A food composition database and a 24 h recall app for dietary intake have been developed for New Caledonian children and adults [75], which have the potential to be adapted for the PNG population. This highlights the need for further investigation into the validation of dietary assessment methods and the need to ensure both social and cultural contextualisation for the PNG population.

This scoping review is the first to summarise the published research on dietary intake among Pacific Islander adults in PNG. The strengths of the study include the use of a robust methodology and the lack of time limits, which allowed for a comprehensive examination of all the literature. The extensive data extracted allowed for energy, nutrients, foods and the context of eating to be described. It also allowed for the dietary behaviours from the different regions within PNG to be highlighted. However, this review is not without limitations. Many papers sought for retrieval were inaccessible due to PNG Medical Journal’s incomplete digital database. Importantly, PNG only gained independence 49 years ago. As one island nation within the Pacific and classified as a LMIC, research within its population is emerging. There is an urgent call for dietary intake research given that the majority of the literature was published before the year 2000, highlighting an important evidence gap. The limited current research on diet in PNG needs to be contextualised, and it may be due to limited resources, capacity and expertise, as well as the complexities of navigating the cultural, geographical, social and political context of doing research in PNG. The rising prevalence of NCDs [63], combined with the nutrition transition [5,7], further emphasises the need to promote and advocate for targeted research activities. There were a range of dietary assessment methods used across studies, as well as inconsistencies in the data reported, such as variation in units of measurement, which limits the comparison of intakes between studies. While some studies used chemical analysis on the foods to determine the nutritional profile, many used a range of existing data sources for foods from different countries. Consequently, the potential for inconsistency and variability in nutrient values across these tables introduces the risk of inaccuracies in evaluating dietary intake. Kaukau is the main food source, and there are over 1000 different varieties [76]. However, its nutrition composition may not be adequately reported for all of the different varieties in food composition databases. There were also inconsistencies in the survey data for some nutrients. The studies mostly reported on grams of macronutrients rather than percentage of energy or gram per kg of body weight. While this study focused only on adults, there is scope for future studies to target infants and children. However, it is important to note that age in such studies is difficult to determine.

## 5. Conclusions

This review highlights an evidence gap in dietary intake research. Within the context of the Healthy Islands initiative, there is an urgent call for this fundamental information, as well as for prioritising funding and resource allocation. It is important that social and cultural contextualisation of dietary behaviours be understood in PNG. The included publications identified a high level of dietary diversity, emphasizing the need for future studies to be region specific. Additionally, the development of a comprehensive PNG food composition database would support various aspects of public health, research, education and policymaking in PNG. This database would be a valuable resource for ensuring accurate and comprehensive information about the nutrient composition of foods consumed in the country. Moreover, creating dietary guidelines in PNG would be beneficial for dietary interventions, supporting prevention of NCDs and assessing the quality of diets, as well as for monitoring changes over time.

## Figures and Tables

**Table 1:** Summary of the study characteristics of included studies on the diets of Pacific Islander adults or households in Papua New Guinea.

First Author, Publication Year, Ref	Study Design	Region/Setting	Population	Data Collection Date	Dietary Assessment Method(s)	Interview Language	Data Source for Foods	Data Reported
Reid, 1969, [22]	NR.	Highlands. Kuru Region, Moke Village, Eastern Highlands Province.	All ages and genders; Individuals (*n* = 38); Lactating (*n* = 2); Pregnant (*n* = 1); Breastfed (*n* = 1). Households (*n* = 3); Household #1 (*n* = 15); Household #2 (*n* = 10); Household #3 (*n* = 13).	1957.	Daily Survey of Diet by Household: 5–7 d household food weighed. Individual Daily Food Consumption: 1 household, for 1 d. The investigator stayed with members of household #3 for the whole day and weighed all the cooked food eaten by each person.	NR.	PNG [36] and foods commonly used in tropical countries [37].	Energy/Nutrients. Foods, Context.
Sinnett, 1973, [23]	Cross-sectional.	Highlands. Murapin tribal community on the hamlet of Tukisenta (Western Highlands).	All ages and genders; Diet survey conducted on individuals (*n* = 90) < 16 y (*n* = 45); 16–65 y (*n* = 45); Average daily dietary intake reported for (*n* = 45) adults. Males (*n* = 23; aged 19–66 y); Females (*n* = 22; aged 16–60 y).	October 1966 to April 1967.	Each household visited 2 times per d (morning and evening meals). All items of food consumed by each individual were weighed for 7 consecutive days. Food eaten away from the house was assessed by weighing a similar portion of the item.	NR.	PNG [38,39], UK [40] and Australia [41].	Energy/Nutrients. Foods, Context.
Harvey, 1983, [24]	NR.	Highlands. Yobakogl Village, Simbu Province.	All ages and genders; Households (*n* = 12); Individuals (*n* = 67); Adult male *(n* = 17); Adult female (*n* = 14); Lactating (*n* = 5). Both genders 15–19 y; (*n* = 6); 10–14 y (*n* = 6); 5–9 y (*n* = 12); 3–4 y (*n* = 2); 1–2 y (*n* = 5).	March 1981.	Each household observed for 5–6 consecutive days by one of seven assistants. Individual intakes were assessed by weighing food consumed and food recalls.	Five assistants able to communicate in the local Sinasina language.	FAO/U.S Department of Health Education and Welfare [42], PNG [43] and foods commonly used in tropical countries [37].	Energy/Nutrients. Foods, Context.
Ohtsuka, 1985, [25]	NR.	Southern. Rual (Northern), Wonie (Inland), Ume (Riverine), and Dorogori (Coastal).	Villages (*n* = 4) Both genders; Individuals (*n* = 132); Rual (1981) (*n* = 26); Wonie (1971) (*n* = 26); Wonie (1981) (*n* = 22); Ume (1981) (*n* = 27); Dorogori (1981) (*n* = 31).	Wonie 1971 (dry season) 1972 (wet season). Four villages in 1981 Rual, Wonie, Ume and Dorogori (dry season).	Each survey recorded amounts of food consumed by 6–8 selected households for 12 (1971–72) and 14 consecutive days (1981). All foods stored in each household were weighed twice (morning and evening).	NR.	Eight food composition tables including PNG and the South Pacific, UK, East Asia and Japan [36,40,42,44,45,46,47,48].	Energy/Nutrients. Foods.
Ulijaszek, 1987, [26]	NR	Southern. Wopkaimin, Western (Fly River) Province. 1975 (Bakonabip hamlet and Tambik and Ulatem hunting shelters). 1984 Atemkit hamlet and Bultem II.	Both genders; 1975: residents varied from 15 to 30, but on one occasion 78 people were present (ritual feast); 1984: all individuals.	1975 and 1984.	1975 hamlet food consumption survey: 47 intake d (male); 47 intake d (female). 1984 weighed dietary intakes 5 d on all individuals in Atemkit hamlet and randomly selected households in Bultem II: 43 intake d (male); 60 intake d (females). Recalls of foods in the absence of recorders.	NR.	PNG [43,49].	Energy/Nutrients. Foods, Context.
Shack, 1990, [27]	Cross sectional.	Momase. Settlers from Grass Country, Wosera/Maprik and Middle Sepik to Gavien, East Sepik Province.	Children and mothers; Families from three ethnic origins within Gavien that had a preschool age child (between 2–6 y) in the family. Total families (*n* = 56); Grass Country (*n* = 20); Wosera (*n* = 18); Middle Sepik (*n* = 18).	November 1985 to September 1986.	24 h recall and frequency of consumption of 16 foods selected to represent a typical diet. Weighed food intakes by a modified method of ‘child following’ determined for a subsample of children and used to verify the accuracy of 24 h recalls. The food frequency list was prepared in PNG and reviewed by the East Sepik Provincial Nutrition Group.	Tok Pisin.	PNG [50].	Energy/Nutrients. Foods, Context.
Grossman, 1991, [28]	NR.	Highlands. Kapanara Village, Kainantu District, Eastern Highlands Province.	Households (*n* = 13); Individuals (*n* = 45); Male (*n* = 23); <10 y (*n* = 3); 10–14 y (*n* = 3); Unmarried adult over 15 y (*n* = 5); Married Adult (*n* = 10); Over 55 y (*n* = 2); Female (*n* = 22); <10 y (*n* = 1); 10–14 y (*n* = 5); Unmarried adult over 15 y (*n* = 2); Married Adult (*n* = 13); Over 55 y (*n* = 1); “Actual amounts” 4 households were part of the food frequency survey.	February to December 1977.	Food frequency interview, considering all foods from previous day. Adults provided dietary data for themselves and children for 69 random/d over 11 m. Children also questioned to confirm the information on meals. Information was also collected on “actual amounts” eaten from a smaller sample of households for 2 consecutive days each month (March to August). Visits were made for all meals during these 2 days. Each cooked food item served to each individual was weighed, including leftovers.	Tok Pisin and interpreters.	NR.	Foods, Context.
Ulijaszek, 1992, [29]	NR.	Southern. Hukim Village, Ningerum, North Fly District, Western (Fly River) Province.	Adult male (*n* = 25) aged 18 and 40 y.	May and November 1984.	Two 5d WFR 6m in-between (wet and wetter season). All food weighed after cooking. Each individual had their own record book. Weights of missing items were estimated by recall using food models. Food eaten outside the home was recorded by weighing where possible, but more often by recall.	NR.	PNG [43], East Asia [51] and South Pacific [52].	Energy/Nutrients. Foods.
Hodge, 1996, [30]	Case-control.	Southern. Wanigela people of Kori, Port Moresby, National Capital District.	Adults (*n* = 285) over 25 y.	August 1991.	24 h recall performed before the questionnaire to identify foods consumed (not in questionnaire). Quantitative FFQ of 87 food and beverage items (allowed for variations in intake associated with season and pay period) from previous 12 m.	NR	NUTTAB 90 nutrient database [53] supplemented with additional information on local food items provided by the Australian Centre for International Agriculture Research, South Pacific Commission and the Papua New Guinea Institute of Medical Research. When data was unavailable, published data was used for similar foods or nutrient analysis performed.	Energy/Nutrients. Foods, Context.
Muntweiler, 2000, [31]	NR.	Highlands. Kainantu and Okapa District. Misapi, Tokai Purosa, Karu 1, Kokopi, Konaka and Norikori villages (Eastern Highlands Province).	Both genders; Households (*n* = 73); (average 6 people per household, 3 adults, 1 school age; 2 below school age per household). Households used as the base unit rather than families as family members do not always live and eat in the same house.	June 1999.	The Agricultural Development Program Nutrition Survey (baseline). Interview conducted in the house with the senior woman from each household and husbands assisted. Questionnaires covered nutrition and protein consumption. Field observations. Following the interview, the house, garden inspected for the types of food crops.	Tok Pisin with discussions in the local languages.	NA.	Foods, Context.
Yamauchi, 2000, [32]	NR.	Highlands. Flat wetland and dry hilly areas of Tari Basin, Southern Highland Province.	Married adults; both genders; Total individuals (*n* = 27); Flat (*n* = 13; *n* = 3 of these lactating); Hilly (*n* = 14; *n* = 1 of these lactating).	June to September 1994.	All foods consumed by each individual throughout the day were weighed before cooking. Participants asked in the morning about the types and amounts of foods consumed during the previous night.	Tok Pisin and interpreter.	Samples of kauaku and green leaves analysed. Other foods PNG and surrounding countries [54,55].	Energy/Nutrients.
Yamauchi, 2001, [33]	NR.	Highlands and Southern. Huli, Tari Basin, Southern Highland Province and Migrants from Tari Basin to Port Moresby, National Capital District.	Married adults (*n* = 56); Rural villagers (*n* = 27); Male (*n* = 15); Female (*n* = 12; including *n* = 4 lactating). Urban migrants (*n* = 29); Male (*n* = 14); Female (*n* = 15; including *n* = 6 lactating). All urban subjects were born and grew up in their homeland in the Tari basin.	July to September 1994 and July to September in 1995.	All foods consumed throughout the day were weighed before cooking. Participants asked in the morning about the types and amounts of foods consumed during the previous night.	Tok Pisin with interpreter.	Samples of kauaku and green leaves analysed. Other foods from PNG and surrounding countries [54,55].	Energy/Nutrients. Context.
Morita, 2015, [34]	NR.	Highlands. Frigano (Eastern Highlands Province), Wenani (Tari Basin, Hela Province) and Levani (Levani Valley, Hela Province).	Adults (*n* = 107); Male: Frigano (*n* = 13); Wenani (*n* = 15); Levani (*n* = 30); Female: Frigano (*n* = 16); Wenani (*n* = 13); Levani (*n* = 20).	February and March 2012–2013.	Semi-quantitative 32-item FFQ. Face-to-face interviews about consumption frequency per day, week or month. Open-ended response questions used for snacks. WFR to evaluate the validity of the FFQ-estimated quantity of an individual’s protein intake.	NR.	PNG [56,57], Australia [58] and Japan [59].	Energy/Nutrients. Foods, Context.
Goris, 2017, [35]	Cross-sectional.	Southern. Kamea, Gulf Province.	Households enrolled in the study (*n* = 70); Infants 0–59 m (*n* = 69); Children 6–12 y (*n* = 151); Non-pregnant women (*n* = 79) from (*n* = 10) villages in Kotidanga Local Level Government.	March to April 2015.	Validated dietary diversity questionnaire, defined as the number of unique foods consumed by household members over a period of time.	NR.	NA.	Foods.

d = days; FFQ = food frequency questionnaire; m = months; NA = not applicable; NR = not reported; PNG = Papua New Guinea; WFR = weighed food record; y = years; 24 h recall = 24 h recall.

**Table 2:** Reported intakes of energy, fibre, protein, carbohydrates, fat and micronutrients for Pacific Islander adults and households in Papua New Guinea.

First Author, Publication Year, Ref	Region	Setting or Year	Gender	Energy (Mean kJ ± SD)	Fibre (g/day)	Protein (g/day ± SD; %E)	Carbohydrate (g/day ± SD; %E)	Fat (g/day ± SD; %E)	Number of Micronutrients Reported
Reid, 1969, [22]	Highlands	Eastern Highlands	Female ^◊^	14,171/day ^§^	-	38	-	-	3 ^a^
			Lactating ^◊^	15,054/day ^§^	-	59	-	-	3 ^a^
		Household #1 (*n* = 15)		329,126/study period ^§^		935 g/study period			3 ^a^
		Household #2 (*n* = 10)		464,089/study period ^§^		1352 g/study period			3 ^a^
		Household #3 (*n* = 13)		343,615/study period ^§^		1185 g/study period			3 ^a^
Sinnett, 1971, [23]	Highlands	Western Highlands	Male	9623/day ^§^	-	25	540	6	-
			Female ^†^	7406/day ^§†^	-	20	410	7	-
Harvey, 1983, [24]	Highlands	Simbu Region 1981	Male	9791 ± 1632/day ^§^	-	43 ± 8; 6%E	-	-	-
			Female	7799 ± 1807/day ^§^	-	36 ± 10; 6%E	-	-	-
			Lactating	7962 ± 870/day ^§^		38 ± 7; 6%E			
Ohtsuka, 1985, [25]	Southern	Rual 1981	Male	14,866/day ^§^	13	54	776	20	-
		Wonie 1971		13,903/day ^§^	16	48	663	41	-
		Wonie 1981		14,853/day ^§^	17	68	785	10	-
		Ume 1981		12,468/day ^§^	12	68	555	54	-
		Dorogori 1981		13,477/day ^§^	10	73	664	25	-
Ulijaszek, 1987, [26]	Southern	1975 (*n* = 47 days intake)	Male	5700 (3760–14,600) ^i^	-	32 (15–72) ^i^	-	3 (1–10) ^i^	5 ^b^
		Non-working 1984 (*n* = 32 days intake)		7480 (5050–10,930) ^i^	-	30 (12–49) ^i^	-	6 (2–32) ^i^	5 ^b^
		Working 1984 (*n* = 11 days intake)		9370 (6740–10,910) ^i^	-	40 (31–60) ^i^	-	22 (11–38) ^i^	5 ^b^
		1975 (*n* = 47 days intake)	Female	5630 (3490–9920) ^i^		29 (15–50) ^i^		3 (1–6) ^i^	5 ^b^
		Non-working 1984 (*n* = 33 days intake)		6240 (4840–8990) ^i^		27 (16–52) ^i^		14 (4–40) ^i^	5 ^b^
		Working 1984 (*n* = 27 days intake)		7300 (9350–9600) ^i^		36 (28–43) ^i^		16 (11–35) ^i^	5 ^b^
Shack, 1990, [27]	Momase	Grass Country	Female	11,820 ± 3699/day ^§^	-	54 ± 29	-	-	-
		Wosera		10,330 ± 3494/day ^§^	-	57 ± 41	-	-	-
		Middle Sepik		9652 ± 4163/day ^§^	-	66 ± 47	-	-	-
Ulijaszek, 1992, [29]	Southern	Rural (Wet)	Male	8660 ± 2680/day ^!^	-	18 ± 5; 3%E	473 ± 126	12 ± 9	7 ^c^
		Rural (Wetter)		8880 ± 2730/day ^!^	-	20 ± 7; 4%E	478 ± 124	15 ± 11	7 ^c^
Hodge, 1996, [30]	Southern	Urban (Port Morsby)	Male	11,500 ± 400/day	22 ± 9	96 ± 32; 14%E	427 ± 159; 59%E	71 ± 28; 23%E	-
			Female	10,500 ± 400/day	21 ± 8	93 ± 34; 15%E	385 ± 155; 58%E	65 ± 27; 23%E	-
Yamauchi, 2000, [32]	Highlands	Flat	Male	15,040 ± 2940/day	-	55 ± 24	-	18 ± 19	-
			Female	13,270 ± 2220/day	-	70 ± 22 62 ± 21 ^‡^	-	31 ± 29	-
		Hilly	Male	9720 ± 3550/day	-	43 ± 35	-	18 ± 24	-
			Female	9140 ± 1570/day	-	37 ± 21 34 ± 17 ^‡^	-	16 ± 18	-
Yamauchi, 2001, [33]	Highlands	Rural	Male	12,230 ± 4200/day	-	49 ± 30	-	18 ± 21	-
			Female	10,530 ± 2350/day	-	48 ± 23 ^‡^	-	23 ± 24	-
	Southern	Urban (Port Moresby)	Male	11,650 ± 2750/day	-	73 ± 21	-	82 ± 48	-
			Female	9620 ± 2650/day	-	65 ± 28 ^‡^	-	84 ± 36	-
Morita, 2015, [34]	Highlands	Frigano	Male	-	-	58 (34–71) ^ii^	-	-	-
			Female	-	-	40 (27–54) ^ii^	-	-	-
		Wenani	Male	-	-	40 (25–60) ^ii^	-	-	-
			Female	-	-	42 (28–59) ^ii^	-	-	-
		Levani	Male	-	-	31 (28–39) ^ii^	-	-	-
			Female	-	-	27 (21–34) ^ii^	-	-	-

All values were rounded to the nearest whole number; ^i^ data formatted with median values (25th and 75th percentiles); ^ii^ data formatted with reported median values (IQR); ^◊^ individual data from Household #3; ^§^ energy converted to kJ from kcal using 4.184; ^†^ data includes ages 16–60 years; ^‡^ adjusted for lactating women; ^!^ between-subject standard deviation; ^a^ thiamine, calcium, vitamin C; ^b^ thiamine, riboflavin, niacin, iron, calcium; ^c^ vitamin A, thiamine, riboflavin, niacin, vitamin C, iron, calcium.

**Table 3:** Food sources contributing to energy and protein, as well as the socio-cultural and economic context of eating for Pacific Islander adults in Papua New Guinea.

First Author, Publication Year, Ref	Region	Population	Food Sources Contributing to Energy	Food Sources Contributing to Protein	Additional Information on Food Sources	Socio-Cultural and Economic Context of Eating
Reid, 1969, [22]	Highlands (Eastern)	Household #1; Day 3	Top 2 foods contributing to energy (kJ) ^§^ Kaukau: 53,375, sugarcane: 6330.	Top 2 foods contributing to protein (g) Kaukau: 77, pitpit (succulent core of a thick-stemmed grass): 60.	“Pigs are plentiful and usually eaten for several meals, at least once a month, and often more frequently”, “gardens are large and always productive”.	Two main meals per day (morning and evening). Mumu (social gatherings).
		Household #1; Day 4	Kaukau: 39,856, sugarcane: 13,209.	Kaukau: 57, ne and ebia (green leaf vegetable): 57.		
Sinnet, 1972, [23]	Highlands (Western)	Adults	Diet consisted almost entirely of kaukau, carbohydrate providing over 90%E.	NR	Fourteen separate varieties of kaukau were consumed by subjects during the survey.	Two main meals per day (morning and evening). Evening meal is a time when family assembles, and friends visit. Influence of cash cropping (cash economy) Social changes in response to European contact.
Harvey, 1983, [24]	Highlands (Simbu)	Adults (1981)	Top 3 food sources (%E) Kaukau: 53, all trade store foods: 26, cereals and grains: 22	Top 3 food sources (%E) Kaukau: 34, all trade store foods: 35, cereals and grains: 25.	The mean number of pigs husbanded by sample households = 4 per year. 51 different varieties of kaukau in the gardens of sample households. Mean daily intake (g) reported as male, female, respectively, for select foods Kaukau: 1107; 875, dark green leaves: 125; 102, pumpkin: 18; 32, corn: 42; 44, rice: 181; 117, flour: 42; 61, tinned fish: 30; 27, sugarcane: 37; 18, pig: 12; 15.	Influence of cash cropping (cash economy).
Ohtsuka, 1985, [25]	Southern Rual (Northern)	Male (1981)	Top 5 food categories contributing to energy intake (kJ) ^§^/day Wild plants: 950, sago: 8000, garden crops: 4079, purchased plants: 858, coconut: 423.	Top 5 food categories contributing to protein intake (g)/day Purchased animals: 55, land animals: 17, garden crops: 12. wild plants: 9, aquatic animals: 8.	NR	NR
	Wonie (Inland)	Male (1971)	Sago: 7644, garden crops: 3841, coconut: 1326, land animals: 586, wild plants: 490.	Purchased animals: 48, land animals: 30, garden crops: 10, sago: 3, coconut: 3.	NR	NR
	Wonie (Inland)	Male (1981)	Garden crops: 6657, sago: 6489, purchased plants: 904, land animals: 749, coconut: 42.	Purchased animals: 68, land animals: 36, garden crops: 23, Purchased plants: 6, coconut 3.	NR	NR
	Ume (Riverine)	Male (1981)	Garden crops: 5176, sago: 2460, purchased plants: 2247, coconut: 1745, aquatic animals: 397.	Purchased animals: 68, garden crops: 17, land animals: 16, aquatic animals: 15, purchased plants: 13.	NR	NR
	Dorogori (Coastal)	Male (1981)	Purchased plants: 5548, garden crops: 5088, sago: 874, coconut: 812, purchased animals: 544.	Purchased animals: 73, purchased plants: 22, aquatic animals: 21, garden crops: 17, land animals: 4.	NR	NR
Ulijaszek, 1987, [26]	Southern (Western)	Bakonabip hamlet 1975	Proportion (%) of dietary energy from foods (top 2) Taro: 67, traditionally reared or hunted animals: 22.	Proportion (%) of dietary protein from foods (top 2) Traditionally reared or hunted animals: 47, taro: 40.	Proportion (%) of dietary energy All plant sources: 78, all animal sources: 22, all store foods: 0 Proportion of dietary protein All plant sources: 53, all animal sources: 47, all store foods: 0.	Influence of cash cropping (cash economy).
		Atemkit hamlet 1984	Kaukau: 46, taro: 35.	Kaukau: 34, traditionally reared or hunted animals: 25.	Proportion (%) of dietary energy All plant sources: 96, all animal sources: 4, all store foods: 0 Proportion of dietary protein All plant sources: 75, all animal sources: 25, all store foods: 0.	
		Bultem II workers 1984	Rice: 22, taro and kaukau: 13.	Tinned meat and fish: 24, rice: 14.	Proportion (%) of dietary energy All plant sources: 80, all store foods: 64, all animal sources: 20 Proportion (%) of dietary protein All store foods: 71, all animal sources: 61, all plant sources: 39. “Western” or imported foods contributed 64% of the energy intake in workers diets and 38% in the diets of workers’ wives”.	
		Atemkit hamlet 1984	Taro: 45, kaukau: 36.	Variety of nuts, fruits and dark leafy greens: 36, taro: 26.	Proportion (%) of dietary energy All plant sources: 99, all animal sources: 1, all store foods: 0 Proportion (%) of dietary protein All plant sources: 91, all animal sources: 9, all store foods: 0	
		Bultem II husbands working 1984	Taro: 31, rice: 28.	Rice: 22, Variety of nuts, fruits and dark leafy greens: 20.	Proportion (%) of dietary energy All plant sources: 90, all store foods: 38, all animal sources: 10 Proportion (%) of dietary protein All plant sources: 63, all store foods: 43, all animal sources: 37.	
Shack, 1990, [27]	Momase	Mothers	NR	NR	Food frequency scores ^†^. Results reported as: Grass Country; Wosera; Middle Sepik Fish (fresh, dried, tinned): 9; 10; 10, rice: 3; 3; 4, coconut: 7; 10; 8, yams + kaukau: 4; 11; 7, greens: 10; 12; 12, sago: 6; 6; 9.	Influence of cash cropping (cash economy)
Grossman, 1991, [28]	Highlands (Eastern)	Household Sample	NR	NR	Most frequently consumed foods, household sample; % of meals in which food is consumed (top 3) Kaukau: 90; pumpkin: 17; enriched rice: 17. Frequency of consumption of food groups, % of meals in which foods appeared Subsistence All subsistence crops: 95 Garden greens: 23, legumes: 12. Purchased Rice, canned fish, canned beef: 19, animal protein: 22, cereal and grains: 20, energy-dense and protein-rich foods: 26.	Two main meals per day (morning and evening). “Villagers ate an average of 2.8 different foods per meal; the morning meal contained an average of 2 foods and the second meal consisted of 3.6 items”. Influence of cash cropping (cash economy).
Ulijaszek, 1992, [29]	Southern (Western)	Adult Males	Proportion of total energy during wet: wetter times of the year (%) Sago: 27:35, plantain: 40:35, starchy roots and tubers: 24:16, rice and wheat products: 2:5, nuts: 3:3, green leafy vegetables: 1:0, all animal foods: 2:5	NR	Pig was shared among kin and only constituted a very small part of the diet.	NR
Hodge, 1996, [30]	Southern (Port Moresby)	Wanigela people of Kori	Percentage contributed by major food sources to energy (%) Cooked banana: 11, white rice: 10, cassava: 7, white bread or roll 6, fish 5, kaukau 4.	Percentage contributed by major food sources to protein (%) Fresh fish: 28, tinned mackerel: 4, tinned corned beef: 4.	Percentage contributed by major food sources to carbohydrate (%) Cooked banana: 17, white rice: 15, cassava: 11, white bread or roll 7, kaukau 6.	Influence of cash cropping (cash economy). Older generations have retained a traditional diet compared to younger generations.
Muntwiler, 2000, [31]	Highlands (Eastern)	Households	NR	Sources of protein foods Winged beans; peanuts; Maggi noodles; lamb flaps; mushroom; wild animals; pig meat; tinned fish; chicken; insects; tinned meat.	% of households Types of foods eaten at breakfast Kaukau: 52, kaukau and greens: 47, sugarcane: 10, pumpkin: 7, corn: 6, tea: 6, taro: 3, potato: 3, beans: 3, chicken: 3, other: 4, nothing: 3. Types of foods eaten at lunch nothing: 63, kaukau: 27, kaukau and greens: 7, sugarcane: 5, sweet banana: 5, fruits: 3, insects: 1. Types of foods eaten at dinner Kaukau and greens: 78, corn: 21, kaukau: 15, potato: 11, rice: 10, tea: 4, chicken: 3, Maggi noodles: 3, pumpkin: 3, banana: 3, taro: 3, other: 9.	Two main meals per day (morning and evening).
Yamauchi, 2001, [33]	Highlands and Southern	Huli, Tari Basin, Southern Highland Province and Migrants from Tari Basin to Port Moresby, National Capital District	NR	NR	NR	Influence of cash cropping (cash economy).
Morita, 2015, [34]	Highlands	Frigano	NR	Median (IQR) protein intake (g) reported by male/female Animal sources: 8 (5–18): 9 (5–14), vegetable sources: 37 (29–62): 31 (22–40), root crops: 8 (4–13): 5 (4–7), green leaves: 5 (3–7): 4 (2–6), rice: 6 (2–16): 4 (1–8), bakery: 5 (1–10): 5 (2–10).	NR	Influence of cash cropping (cash economy).
		Wenani	NR	Animal sources: 7 (2–20): 6 (2–9), vegetable sources: 32 (24–40): 38 (23–49), root crops: 15 (13–17): 15 (14–19), green leaves: 3 (2–5): 3 (2–3), rice: 3 (1–7): 4 (1–6), bakery: 2 (1–9): 3 (1–6).	NR	
		Levani	NR	Animal sources: 4 (2–8): 5 (1–9), vegetable sources: 26 (23–31): 21 (15–27), root crops: 16 (14–20): 12 (10–15), green leaves: 2 (1–5): 1 (0–3), rice: 3 (1–5): 2 (1–3), bakery: 0 (0–0): (0–0).		
Goris, 2017, [35]	Southern (Gulf)	Infants, Children, and non-pregnant women	NR	NR	% of households consuming the food group White roots and tubers: 100, dark green leafy vegetables: 100, betel nut, tobacco: 86, oils, fats, butter, coconut: 74, vegetables, tubers fruits that are yellow or orange: 41, legumes, nuts, seeds: 29, flesh meat: 29, grubs, snails or insects: 7, other fruits or vegetables: 6, eggs: 6, fish (tinned): 6, foods made from grains: 4, sugary foods: 3, organ meat: 0, milk (powder), cheese, yoghurt products: 0.	NR

Values rounded to nearest whole number; NR = not reported; kaukau = sweet potato; pitpit = succulent core of a thick-stemmed grass; ebia = green leaf vegetable; ne = green leaf vegetable: ^§^ energy converted to kJ from kcal using 4.184; ^†^ participants asked how often each food was consumed in the previous three months and intake was scored: never = 0, once per month = 1, twice per month = 2, three to four times per month = 4, twice per week = 8 and three or more times per week = 12. The maximum frequency score was 12.

## Supplementary Materials

The following supporting information can be downloaded at: https://www.mdpi.com/article/10.3390/nu16101472/s1, Table S1: Medline search; Table S2: Detailed summary on dietary assessment methods and data collection.

## References

[1] The World Bank (2024). World Development Indicators. https://databank.worldbank.org/reports.aspx?source=2&country=PNG.

[2] Government of Papua New Guinea (2021). National Health Plan 2021–2030. 1a Policies and Strategies.

[3] University of Hawai‘i at Mānoa Nutrition Transition of the Pacific. https://manoa.hawaii.edu/ctahr/pacificfoodguide/index.php/about-the-guide/nutrition-transition-of-the-pacific/#:~:text=Nutrition%20transition%20refers%20to%20the,Gordon%2DLarsen%2C%202004)..

[4] Walls H.L., Johnston D., Mazalale J., Chirwa E.W. (2018). Why we are still failing to measure the nutrition transition. BMJ Glob. Health.

[5] Schmidt E., Fang P. (2021). Papua New Guinea agri-food trade and household consumption trends point towards dietary change and increased overweight and obesity prevalence. Glob. Health.

[6] Conn C., Camnmock R., Ford K., Faesen Kloet G., Nayar S. (2020). Our people, our food, our planet: Sustainable food systems policy in the Pacific. Pac. Health.

[7] Rarau P., Vengiau G., Gouda H., Phuanukoonon S., Kevau I.H., Bullen C., Scragg R., Riley I., Marks G., Umezaki M. (2017). Prevalence of non-communicable disease risk factors in three sites across Papua New Guinea: A cross-sectional study. BMJ Glob. Health.

[8] World Bank (2022). New World Bank Country Classifications by Income Level: 2022–2023. https://blogs.worldbank.org/en/opendata/new-world-bank-country-classifications-income-level-2022-2023.

[9] World Health Organization (2014). Global Status Report on Noncommunicable Diseases 2014.

[10] World Health Organization (2011). Global Status Report on Noncommunicable Diseases 2010.

[11] World Health Organization (2023). Noncommunicable Diseases. https://www.who.int/news-room/fact-sheets/detail/noncommunicable-diseases.

[12] World Cancer Research Fund Mouth and Oral Cancers. https://www.wcrf.org/diet-activity-and-cancer/cancer-types/mouth-pharynx-larynx-cancers/#:~:text=Smoking%20(or%20the%20use%20of,of%20the%20mouth%20and%20pharynx.

[13] World Health Organization (2024). Pacific Health Ministers Meetings. https://www.who.int/westernpacific/about/how-we-work/pacific-support/pacific-health-ministers-meetings.

[14] World Health Organization (2015). The First 20 Years of the Journey towards the Vision of Healthy Islands in the Pacific. https://www.who.int/publications/i/item/9789290617150.

[15] Papua New Guinea National Nutrition Policy 2016–2026. https://faolex.fao.org/docs/pdf/PNG182140.pdf.

[16] Department of Agriculture and Livestock Papua New Guinea National Food Security Policy 2018–2027. https://faolex.fao.org/docs/pdf/png202059.pdf.

[17] Munn Z., Peters M.D.J., Stern C., Tufanaru C., McArthur A., Aromataris E. (2018). Systematic review or scoping review? Guidance for authors when choosing between a systematic or scoping review approach. BMC Med. Res. Methodol..

[18] Peters M.D.J., Marnie C., Tricco A.C., Pollock D., Munn Z., Alexander L., McInerney P., Godfrey C., Khalil H. (2020). Updated methodological guidance for the conduct of scoping reviews. JBI Evid. Synth..

[19] Tricco A.C., Lillie E., Zarin W., O’Brien K.K., Colquhoun H., Levac D., Moher D., Peters M.D., Horsley T., Weeks L. (2018). PRISMA Extension for Scoping Reviews (PRISMA-ScR): Checklist and Explanation. Ann. Intern. Med..

[20] Page M.J., E McKenzie J., Bossuyt P.M., Boutron I., Hoffmann T.C., Mulrow C.D., Shamseer L., Tetzlaff J.M., A Akl E., E Brennan S. (2021). The PRISMA 2020 statement: An updated guideline for reporting systematic reviews. BMJ.

[21] Arksey H., O’Malley L. (2005). Scoping studies: Towards a methodological framework. Int. J. Soc. Res. Methodol..

[22] Reid L.H., Gajdusek D.C. (1969). Nutrition in the kuru region. 2. A nutritional evaluation of traditional Fore diet in Moke village in 1957. Acta Trop..

[23] Sinnet P.F., Whyte H.M. (1973). Epidemiological Studies in a Highland Population of New Guinea: Environment, Culture, and Health Status. Hum. Ecol..

[24] Harvey P.W., Heywood P.F. (1983). Twenty-five years of dietary change in Simbu province, Papua New Guinea. Ecol. Food Nutr..

[25] Ohtsuka R., Inaoka T., Kawabe T., Suzuki T., Hongo T., Akimichi T. (1985). Diversity and Change of Food-Consumption and Nutrient Intake among the Gidra in Lowland Papua. Ecol. Food Nutr..

[26] Ulijaszek S.J., Hyndman D.C., Lourie J.A., Pumuye A. (1987). Mining, Modernization and Dietary Change among the Wopkaimin of Papua-New-Guinea. Ecol. Food Nutr..

[27] Shack K.W., Grivetti L.E., Dewey K.G. (1990). Cash Cropping, Subsistence Agriculture and Nutritional Status among Mothers and Children in Lowland Papua New Guinea. Soc. Sci. Med..

[28] Grossman L.S. (1991). Diet, Income, and Subsistence in an Eastern Highland Village, Papua-New-Guinea. Ecol. Food Nutr..

[29] Ulijaszek S.J. (1992). Dietary and nutrient intakes of 25 Ningerum (New Guinea) adult males at two times of the year. Am. J. Hum. Biol..

[30] Hodge A.M., Montgomery J., Dowse G.K., Mavo B., Watt T., Alpers M.P., Zimmet P.Z. (1996). Diet in an urban Papua New Guinea population with high levels of cardiovascular risk factors. Ecol. Food Nutr..

[31] Muntwiler M., Shelton R.M. (2000). Survey of nutrition and protein intake in rural families in Eastern Highlands Province. Food Security for Papua New Guinea, Proceedings of the Papua New Guinea Food and Nutrition 2000 Conference, Lae, Papua New Guinea, 26–30 June 2000.

[32] Yamauchi T. (2000). Impact of microenvironment on food security and nutritional adaptation in the Tari basin. Food Security for Papua New Guinea, Proceedings of the Papua New Guinea Food and Nutrition 2000 Conference, Lae, Papua New Guinea, 26–30 June 2000.

[33] Yamauchi T., Umezaki M., Ohtsuka R. (2001). Influence of urbanisation on physical activity and dietary changes in Huli-speaking population: A comparative study of village dwellers and migrants in urban settlements. Br. J. Nutr..

[34] Morita A., Natsuhara K., Tomitsuka E., Odani S., Baba J., Tadokoro K., Igai K., Greenhill A.R., Horwood P.F., Soli K.W. (2015). Development, validation, and use of a semi-quantitative food frequency questionnaire for assessing protein intake in Papua New Guinean Highlanders. Am. J. Hum. Biol..

[35] Goris J.M., Zomerdijk N., Temple V.J. (2017). Nutritional status and dietary diversity of Kamea in Gulf Province, Papua New Guineas. Asia Pac. J. Clin. Nutr..

[36] Hodges K., Fysh C.F., Rienits K. (1947). New Guinea and Papuan food composition tables. Report of the New Guinea Nutrition Survey Expedition.

[37] Platt B.S. (1962). Tables of representative values of foods commonly used in tropical countries. Tables of Representative Values of Foods Commonly Used in Tropical Countries.

[38] Hipsley E.H., Clements F.W. (1947). Report of the New Guinea Nutrition Survey Expedition.

[39] Hipsley E.H., Kirk N.E. (1965). Studies of Dietary Intake and the Expenditure of Energy by New Guineans.

[40] McCance R., Widdowson E. (1960). The Composition of Foods-Medical Research Council Special.

[41] Osmond A. (1948). Tables of Composition of Australian Foods.

[42] FAO/USDHEW (1972). Food Composition Table for Use in East Asia.

[43] Norgan N., Durnin J., Ferro-Luzzi A. (1979). The Composition of Some New Guinea Foods. Papua N. Guin. Agric. J..

[44] Bailey K. (1968). Composition of New Guinea Highland Foods.

[45] Murai M., Miller C.D., Pen F. (1958). Some Tropical South Pacific Island Foods: Description, History, Use, Composition, and Nutritive Value.

[46] Cresta M., Allegrini M., Casadei E., Gallorini M., Lanzola E., Panatta G. (1976). Benin: Nutritional considerations on trace elements in the diet. Food Nutr..

[47] Resources Council (1982). Standard Tables of Food Composition in Japan.

[48] Gormican A. (1970). Inorganic elements in foods used in hospital menus. J. Am. Diet. Assoc..

[49] Dornstreich M.D. (1973). An Ecological Study of Gadio Enga (New Guinea) Subsistence.

[50] Groos A., Ulijaszek S., Heywood P. (1986). Food Tables for Use in Papua New Guinea.

[51] National Institute of Arthritis M., Diseases D., Leung W.-T.W., Butrum R.R., Chang F.H. (1973). Food Composition Table for Use in East Asia.

[52] Peters F. (1959). Chemical composition of South Pacific foods. Qual. Plant. Et Mater. Veg..

[53] Department of Community Services and Health (1990). NUTTAB 90 Nutrient Data Table for Use in Australia.

[54] Hongo T., Ohtsuka R. (1993). Nutrient composition of Papua New Guinea foods. Man Cult. Ocean..

[55] Umezaki M., Yamauchi T., Ohtsuka R. (1998). Diet among the Huli in Papua New Guinea Highlands when they were influenced by the extended rainy period. Ecol. Food Nutr..

[56] Umezaki M., Natsuhara K., Ohtsuka R. (2001). Protein content and amino acid scores of sweet potatoes in Papua New Guinea Highlands. Ecol. Food Nutr..

[57] Dignan C.B.B., Kumar S., Aalbersberg W. (2004). The Pacific Islands Food Composition Tables.

[58] Food Standards Australia New Zealand Australian Food, Supplement & Nutrient Database 2007. https://www.foodstandards.gov.au/science-data/monitoringnutrients/ausnut/ausnut2007.

[59] Japanese Ministry of Education, Culture, Sports, Science and Technology (2005). Standard Tables of Food Composition in Japan. https://www.mext.go.jp/en/policy/science_technology/policy/title01/detail01/1374030.htm.

[60] National Health and Medical Research Council Nutrient Refernece Values for Australia and New Zealand. https://www.eatforhealth.gov.au/nutrient-reference-values/chronic-disease/macronutrient-balance.

[61] Centers for Disease Control and Prevention (2022). Poor Nutrition. https://www.cdc.gov/chronicdisease/resources/publications/factsheets/nutrition.htm#:~:text=Adults%20who%20eat%20a%20healthy,these%20conditions%20and%20avoid%20complications.

[62] Vengiau G. (2019). Nutrition Transition in Papua New Guinea (PNG): An Assessment of the Nutrition Transition for Three Diverse Populations, Including the Contributing Factors, Food Insecurity, and Health Risks University of Queensland.

[63] Rarau P., Guo S., Baptista S.N., Pulford J., McPake B., Oldenburg B. (2020). Prevalence of non-communicable diseases and their risk factors in Papua New Guinea: A systematic review. SAGE Open Med..

[64] Peng W., Zhang L., Wen F., Tang X., Zeng L., Chen J., Galea G., Wen D., Wang Y. (2023). Trends and disparities in non-communicable diseases in the Western Pacific region. Lancet Reg Health West Pac..

[65] World Health Organization (2020). For the Future towards the Healthiest and Safest Region. https://iris.who.int/bitstream/handle/10665/330703/WPR-2020-RDO-001-eng.pdf?sequence=1.

[66] United Nations The 17 Goals. https://sdgs.un.org/goals.

[67] Perna M., Hewlings S. (2022). Saturated Fatty Acid Chain Length and Risk of Cardiovascular Disease: A Systematic Review. Nutrients.

[68] Rarau P., Pulford J., Gouda H., Phuanukoonon S., Bullen C., Scragg R., Pham B.N., McPake B., Oldenburg B. (2019). Socio-economic status and behavioural and cardiovascular risk factors in Papua New Guinea: A cross-sectional survey. PLoS ONE.

[69] Gobal Nutrition Report (2024). Country Nutrition Profiles. https://globalnutritionreport.org/resources/nutrition-profiles/oceania/melanesia/papua-new-guinea/#:~:text=28.9%25%20of%20adult%20(aged%2018,women%20and%2030.4%25%20for%20men.

[70] Ministry of Health National Policy on Health Promotion For Papua New Guinea. https://docplayer.net/22960586-National-policy-on-health-promotion-for-papua-new-guinea.html.

[71] Atoloye A.T., Savoie-Roskos M.R., Guenther P.M., Durward C.M. (2021). Effectiveness of Expanded Food and Nutrition Education Program in Changing Nutrition-Related Outcomes Among Adults With Low Income: A Systematic Review. J. Nutr. Educ. Behav..

[72] Andrew M., Barker P.J. (2022). Lessons learned on School Agriculture and Nutrition in Papua New Guinea.

[73] Food and Agriculture Organisation of the United Nations Food-Based Dietary Guidelines. https://www.fao.org/nutrition/education/food-based-dietary-guidelines.

[74] World Health Organization Regional Office for the Western Pacific (1999). Development of Food-Based Dietary Guidelines for the Western Pacific Region: The Shift from Nutrients and Food Groups to Food Availability, Traditional Cuisine and Modern Foods in Relation to Emerging Chronic Noncommunicable Diseases.

[75] Chen J., Bertrand S., Galy O., Raubenheimer D., Allman-Farinelli M., Caillaud C. (2021). The Design and Development of a Food Composition Database for an Electronic Tool to Assess Food Intake in New Caledonian Families. Nutrients.

[76] Chang H., Villano R., Irving D., Kewa L., Mais A. (2017). Understanding Consumer Preferences for Sweetpotato in Papua New Guinea. Australas. Agribus. Perspect..

