# Supplementary material for: What Do We Know about the Diets of Pacific Islander Adults in Papua New Guinea? A Scoping Review
Source: Nutrients. 2024 May 13;16(10):1472. doi: 10.3390/nu16101472 (PMC11124261; doi:10.3390/nu16101472)
Supplement: Supplementary file 1 [file nutrients-16-01472-s001.zip › nutrients-3005554-supplementary.pdf]

Supplementary Table S1. Medline search conducted on the 01 March 2023.

#	Query	Result
1	exp Papua New Guinea/	
2	exp "Native Hawaiian or Other Pacific Islander"/	
3	papua*.tw. OR New Guinea*.tw. OR PNG.tw. OR Pacific Island*.tw. OR Pacific region*.tw. OR Melanesian*.tw. OR Port Moresby.tw. OR National Capital District.tw.	
4	OR 1 OR 2 OR 3	32,173
5	"diet, food, and nutrition"/ or diet/	
6	"Energy intake*".tw. OR Calori*.tw.OR Megajoule*.tw. OR Kilojoule*.tw. OR Joule*.tw. OR "Energy dens*".tw. OR "Nutrient dens*".tw. OR kcal*.tw. OR kJ.tw. OR Nutri*.tw. OR M?cronutrient*.tw. OR Protein*.tw. OR Fat.tw. OR Fats.tw. OR Carbohydrate*.tw. OR carbs.tw. OR Carb.tw. OR CHO.tw. OR alcohol*.tw. OR Vitamin*.tw. OR Mineral*.tw. OR Fiber.tw. OR Fibre.tw. OR Food*.tw. OR Kai Kai.tw. OR "Sweet potato*".tw. OR Taro.tw. OR Cassava.tw. OR Yam*.tw OR Sago.tw. OR Breadfruit.tw. OR "Leafy green*".tw. OR Kumu.tw. OR Coconut*.tw. OR Banana*.tw. OR Guava*.tw. OR Pineapple*.tw. OR Watermelon*.tw. OR Papaya*.tw. OR Mango*.tw. OR "Core Foods*".tw. OR "Five Food Group*".tw. OR Vegetable*.tw. OR Legume*.tw. OR Bean*.tw. OR Grain*.tw. OR Cereal*.tw. OR Wholegrain*.tw. OR Fruit*.tw. OR "Lean meat*".tw. OR Poultry.tw. OR Fish*.tw. OR Seafood.tw. OR Sea food.tw. OR Egg*.tw. OR Tofu.tw. OR Nut*.tw. OR Seed*.tw. OR Dairy.tw. OR Milk.tw. OR Yoghurt.tw. OR Cheese.tw. OR Snack*.tw. OR Discretionary food*.tw. OR "Miscellaneous food*".tw. OR "Convenience food*".tw. OR "Junk food*".tw. OR "Fast food*".tw. OR "Processed food*".tw. OR "Processed products".tw. OR "Ultraprocessed food*".tw. OR "Ultra processed food*".tw. OR "High fat*".tw. OR "High sugar*".tw. OR "Fried food*".tw.	
7	Beverage*.tw. OR ((Sugar sweetened or sugar* or sweet* or fruit* or flovoured or carbonated) adj (beverage* or drink*)).tw. OR SSB.tw. OR (Flavoured adj (dairy* or milk*)).tw. OR (Drink* adj (soft or fizzy or energy)).tw. OR Soda.tw. OR Pepsi.tw. OR Cola.tw. OR Coke.tw. OR Lemonade.tw.	
8	Nutritional status/	
9	Nutrition Assessment/	
10	Diet Records/	

11	Nutrition Surveys/	
12	Diet Surveys/	
13	(nutri* adj3 (questionnaire* or assess* or survey* or record* or recall* or method* or 24-h* or image* or app* or diar* or data or intervention* or quality or intake* or traditional or village* or coastal or highland* or health* or consumption)).tw.	
14	(diet* adj3 (questionnaire* or assess* or survey* or record* or recall* or method* or 24-h* or image* or app* or diar* or data or intervention* or quality or intake* or traditional or village* or coastal or highland* or vegetarian or pattern*)).tw.	
15	(food adj3 (questionnaire* or assess* or survey* or record* or recall* or method* or 24-h* or image* or app* or diar* or data or intervention* or quality or intake* or traditional or village* or coastal or highland* or vegetarian or pattern* or consumption)).tw.	
16	OR 5 OR 6 OR 7 OR 8 OR 9 OR 10 OR 11 OR 12 OR 13 OR 14 OR 15	7126026
16	4 AND 16	5862

Supplementary Table S2. Detailed summary on dietary assessment methods and data collection. Papua New Guinea

First author, publication year	Dietary assessment method(s)	Additional information: data collection
**Reid, 1969**	Daily Survey of Diet by Household: 5-7day household food weighed. Each day the women went to the gardens in the morning and returned at sundown with food for the household (enough for the evening and morning meal). The women were met by the investigator as they returned from the gardens. The investigator sorted and weighed the food (raw). Food remaining in the house from the previous day was weighed (mostly kaukau). From day 2, individuals were questioned about daily snacks and amounts estimated.<br><br>Individual Daily Food Consumption: 1 household, for 1 day. The investigator stayed with members of household #3 for the whole day and weighed all the food eaten by each person (cooked).	• Considerable change in the individuals who ate at each household from day to day.<br>• Household members would visit elsewhere for meals, so names and days eating in household were recorded.<br>• Transients who ate ~half their full daily diet in the household were recorded.<br>• Meals are prolonged over several hours, during which time food is being continually cooked and eaten.<br>• The different types of greens were classified (if possible), but several varieties are usually picked, mixed and cooked together.<br>• The edible proportion of pitpit estimated as one half of that brought from the garden.<br>• Loss in weight of root vegetables during cooking and the discarding of the skin before eating was not taken into account in the Household Survey, but prepared food was weighed in the Daily Individual Survey.<br>• The weight of foods was obtained in lbs and ozs and converted to grams for the analysis.<br>• The greatest proportion of greens eaten consisted of ebia of which no nutritive analysis could be found. Therefore, nutrient analysis of ebia and most other greens estimated from data for "high carotene dark green leaves" from the New Guinea and Papuan Food Composition.

First author, publication year	Dietary assessment method(s)	Additional information: data collection
		• Many foods were matched to similar foods e.g. wild birds matched to squab (pigeon).
**Sinnett, 1973**	Each household visited 2 times per day (morning and evening meals). All items of food consumed by each individual were weighted for 7 consecutive days. Food eaten away from the house was assessed by weighing a similar portion to the item.	• Family groups (considered to comprise of a husband, his wives and their unmarried children).<br>• Age is difficult to determine in such populations and subject to systematic errors.<br>• Chemical composition of kaukau calculated on the basis of fresh wet weight and then applied to the weight of cooked food consumed (the values may underestimate the intake by about 15% due to water loss).<br>• Significant differences in chemical composition exist among varieties of kaukau.
**Harvey, 1983**	Each household observed for 5-6 consecutive days by one of seven assistants. Individual intakes were assessed by weighing food consumed and food recalls. All food produced or purchased were recorded. Assistants waited at the house until return of the days harvest. All food weighed and intended use recorded. Each household member asked to recall any food eaten during the day and quantities estimated compared to samples. Food weighed before preparation and before portioned to individuals. The assistant stayed until the evening meal was finished and they commenced again at sunrise. The 24 hour period finished with completion of the morning meal. During the day (when possible) the assistants accompanied adults to the garden. During these visits the assistants observed and weighed any snacks and recorded all crops and varieties of kaukau. Effort to not disrupt normal eating habits of the household. Difficulties with composite dishes (in such cases, the ingredients were weighed before preparation and the weights of ingredients served to a particular person calculated in proportion to the weight of the individual's meal).	• Potential data collection errors include: "estimation of quantities of food not observed, variability of weight changes in the preparation of food, variability of nutrient composition of crops with variety and growing conditions, and some observer effects"
**Ohtsuka, 1985**	Each survey recorded amounts of food consumed by 6-8 selected households for 12 (1971-72) and 14 consecutive days (1981). All foods stored in each household were weighed	• When members of households stayed out of the village overnight for a period of time (the days were excluded).<br>• Sometimes failed to weigh food prior to eating.

First author, publication year	Dietary assessment method(s)	Additional information: data collection
	twice (morning and evening). All food flows from and to the household during the period were measured. All food weighed just prior to cooking or eating. Store foods weighed to check reduced amounts.	• Difficult to be present each time cooking or snacking occurred (therefore cooked foods were weighed and households questioned about the foods eaten).<br>• When people outside the village consumed food that was obtained or harvested elsewhere, this resulted in estimations.<br>• When village-mates joined the meal and some household members did not have the meal, the amounts of foods consumed were adjusted by applying man-value coefficient.
**Ulijaszek, 1987**	1975 hamlet food consumption survey: 47 intake days (male); 47 intake days (female). Daily intakes of different foods were estimated by subtracting wastage from the amount of food entering the hamlet or shelter, then dividing by the number of individuals. Allowances for different portion sizes between children/adults were made.<br><br>1984 weighed dietary intakes 5 days on all individuals in Atemkit hamlet and randomly selected households in Bultem II: 43 intake days (male); 60 intake days (females)<br><br>All foods weighed after cooking in the morning and evening by 2 authors. Those who remained at the location during the day also had their food weighed. Recalls of foods in the absence of recorders. Individuals with less than two-thirds of their total daily food consumption weighed were excluded. If more than 2 days were excluded, all data excluded.	• Number of residents in these locations varied from day to day.<br>• Water weight of stable foods tested in the laboratory and corrected.<br>• Report not from a single coordinated study but several studies using different sampling methods.
**Shack, 1990**	24 hour recall and frequency of consumption of 16 foods selected to represent a typical diet.<br><br>Only 24 hour recall collected for children. Mother completed two 24 hour recalls, one for her and one for her child. Amounts consumed were recorded with reference to the eating utensils used in the home and later converted to grams.	• Visit of household gardens to determine the distance from the house, size and number of crops grown.<br>• Food frequency list underestimates foods such as sago which is consumed twice daily by many individuals.<br>• The food frequency data presented are for staple foods which consumption does not vary greatly from month to month or seasonally (with possible exceptions being yams and kauakau).<br>• Nutrient adequacy score was computed for each mother and child for diet quality.

First author, publication year	Dietary assessment method(s)	Additional information: data collection
	Weighed food intakes by a modified method of 'child following' determined for a subsample of children and used to verify the accuracy of 24 hour recalls.<br><br>The food frequency list was prepared in Papua New Guinea and reviewed by the East Sepik Provincial Nutrition Group; 'how often each food was consumed in the previous 3months' and intakes scored.	
**Grossman, 1991**	Food frequency interview, considering all foods from previous day. Adults provided dietary data for themselves and children for 69 random/days over 11months. Children also questioned to confirm the information on meals.<br><br>Information was also collected on "actual amounts" eaten from a smaller sample of households for 2 consecutive days each month (March to August). Visits were made for all meals during these 2 days. Each cooked food item served to each individual were weighed, including leftovers (where possible). If snacks were consumed away from home, amounts were estimated using reference foods. Not always possible to collect dietary data of all members of the "actual amounts" households for a variety of reasons (e.g being absent from the village).	• Memory of food recall, particularly snacks (minimised through probing).<br>• Sugar cane and cucumbers consumed in the gardens likely to be under-represented (related primarily to subsistence foods).<br>• Data on some seasonal subsistence foods eaten early in the year may not be fully representative of the actual frequency of consumption.<br>• Sample members considered as having 2 meals per day (morning and rest of the day).<br>• Less frequently, their main meal is in the afternoon (e.g. at ceremonial events).<br>• Snacks sometimes eaten in the afternoon but accurately distinguishing what was eaten in the afternoon from later in the evening was not possible. Food consumed in the afternoon and evening was considered a single meal.<br>• Heavy consumption of beer during coffee-harvesting season resulted in inaccurate information on their previous days diet (therefore data not recorded). Not possible to obtain accurate data on the frequency of beer drinking.<br>• Incomes for 10 households headed by married men (Papua New Guinea Kina 123-988) and 3 households had minimal incomes (Papua New Guinea Kina14-43). Analyses included consumption of purchased foods (data from 10 households as minimal income would bias results for dietary patterns) and 13 households (for subsistence foods).
**Ulijaszek, 1992**	Two 5 day Weighed Food Record 6 months in between (wet and wetter season). All food weighed after cooking. Each individual had their own record book. Weights of missing items were estimated by recall using food models. Food eaten outside the home was recorded by weighing where possible, but more often by recall.	• Consumed meals out of home were weighed where possible, but more frequently through recall.<br>• Individuals with less than two-thirds of their total daily food consumption weighed (excluded from the analysis n=12).<br>• To reduce error of water content of staple foods, samples were collected and water weight determined by drying to a constant weight.<br>• Correction factors between measured and literature values applied.

First author, publication year	Dietary assessment method(s)	Additional information: data collection
**Hodge, 1996**	24 hour recall performed before the questionnaire to identify foods consumed (not in questionnaire).<br><br>Quantitative Food Frequency Questionnaire of 87 food and beverages (allowed for variations in intake associated with season and pay period) from previous 12 months.	• Food Frequency Questionnaire included ethnic and regional foods.<br>• Questions regarding alcohol not included (most Seventh Day Adventists).<br>• Quantities relative to 'standard serves' estimated using standard weighed samples of staple foods along with food models, reference portion sizes on common canned/bottled foods and household measures.<br>• For each item, the subjects usual serving size relative to the standard was recorded.<br>• Frequency categories not specified on the questionnaire, leaving interviewers to determine the appropriate frequency.<br>• Seasonal foods identified and intake over a specified time recorded.<br>• Details around cooking methods, types/amounts of fat, milk, sugar, takeaway and snacks recorded.<br>• Open ended questions asked to recall intake of frequently consumed foods not on the questionnaire.
**Muntweiler, 2000**	The Agricultural Development Program Nutrition Survey (baseline). Interview conducted in the house with the senior woman from each household and husbands assisted. Questionnaires covered nutrition and protein consumption.<br><br>Field observations. Following the interview, the house garden inspected for the types of food crops. The number of trees were counted and recorded.	• The Agricultural Development Program Nutrition Survey is based on a questionnaire used in the Papua New Guinea National Nutrition Survey of 1882-83.<br>• Broad comparisons made between the two surveys, but the National Nutrition Survey is district level, not at the level of individual villages.<br>• Quantities defined in broad categories (e.g small/large amounts). Terms (e.g. previous day/previous week) to determine when events occurred instead of asking the interviewees for the name of weekdays or dates.
**Yamauchi, 2000 and 2001**	All foods consumed throughout the day for each individual were weighed before cooking. Participants asked in the morning about the types and amounts of foods consumed during the previous night.	• Participants might eat less on the day of investigation because of constant observation.

First author, publication year	Dietary assessment method(s)	Additional information: data collection
**Morita, 2015**	Semi-quantitative 32-item Food Frequency Questionnaire. Face-to-face interviews about consumption frequency per day, week or month. Open-ended response questions used for snacks. Usual portion sizes specified using realistic food samples for root crops, animals and leafy vegetables, body parts for fish and natural units (e.g. piece, plate, small/medium/large) for other food items.<br><br>For commonly shared food (rice, noodles, canned fish/meat) an adjustment was made where the median portion ratio for male:female to be 1.5:1<br><br>Weight Food Record to evaluate the validity of the Food Frequency Questionnaire estimated quantity of an individuals protein intake.	• Interpretation of natural units varied from site to site, so samples gathered from markets and gardens in each site and the average used as the usual portion.<br>• The food items were classified into animal or vegetable source.<br>• Mixed food was classified depending on the main ingredient.<br>• The food items were also classified into subsistence food or store-bought.
**Goris, 2017**	Validated dietary diversity questionnaire defined as the number of unique foods consumed by household members over a period of time.	• Children excluded if age could not be determined.<br>• Females ages based on self-report or estimated by using local events calendar.
